# Sonographic Signatures of Immune Checkpoint Inhibitor-Associated Musculoskeletal Adverse Events

**DOI:** 10.3390/cancers17142344

**Published:** 2025-07-15

**Authors:** Hans Vitzthum von Eckstaedt, Kevin Weng, Ingeborg Sacksen, Rachael Stovall, Petros Grivas, Shailender Bhatia, Evan Hall, Scott Pollock, Namrata Singh

**Affiliations:** 1 Division of Rheumatology, University of Washington, Seattle, WA 98195, USAsackseni@uw.edu (I.S.); stovall@uw.edu (R.S.); pspolloc@uw.edu (S.P.); 2 School of Public Health, University of Washington, Seattle, WA 98195, USA; kevchenn@uw.edu; 3 Division of Hematology-Oncology, University of Washington, Seattle, WA 98109, USA; pgrivas@uw.edu (P.G.); sbhatia@uw.edu (S.B.); evanh@uw.edu (E.H.); 4 Department of Epidemiology, University of Washington, Seattle, WA 98195, USA

**Keywords:** immune-related adverse events, immunotherapy, musculoskeletal ultrasound, diagnostic tool, melanoma

## Abstract

**Background**: Immune checkpoint inhibitors (ICIs) transformed cancer treatment, producing significant survival benefits. However, ICIs can trigger toxicities called immune-related adverse events (irAEs), including inflammatory arthritis (IA) and polymyalgia rheumatica (PMR)-like syndromes. Our study aimed to systematically further characterize musculoskeletal ultrasound (MSKUS) findings in patients with ICI-IA and ICI-PMR, collectively referred to as “MSK-irAEs”, and explore the role of US in guiding treatment. **Methods**: The authors conducted a comprehensive chart review for patients receiving ICIs undergoing MSKUS at our center’s rheumatology clinics. US examinations were performed and reviewed by two MSKUS-certified rheumatologists. Descriptive statistics were performed to summarize demographic, clinical, and treatment-related variables. US findings were categorized with a novel scoring system: 0—no signs of inflammatory arthropathy or tendinopathy, 1—potential signs of inflammation (grayscale ≥ 2, effusion without power Doppler, synovial hypertrophy in the joint), and 2—active inflammation in joints and/or tendons (characterized by power Doppler) and signs of inflammation. **Results**: Twenty-three patients were included. The median age was 63 years, 52% were male, and 87% were White. Melanoma was the most common cancer (48%). MSK-irAEs were diagnosed in nineteen (83%), with MSKUS showing inflammation in seventeen (74%). Sixteen (70%) received escalation in MSK-irAE treatment after MSKUS. Four (17%) had erosive disease due to MSK-irAEs, while one had erosive osteoarthritis. Individuals with inflammatory erosive changes experienced prolonged intervals between symptom onset and MSKUS, ranging from 17 to 82 months, suggesting that erosions may reflect chronic, under-recognized inflammation. On MSK-irAE therapy, nine (47%) experienced symptomatic improvement, five (26%) achieved resolution, and in four (21%) cases, it was too early to assess the response. MSKUS detected other causes of MSK symptoms besides MSK-irAEs in several patients, allowing ICI resumption in one. **Conclusions**: Our study highlights the clinical utility of MSKUS not only as a diagnostic tool but also to guide therapeutic decision-making.

## Simple Summary

Immune checkpoint inhibitors (ICIs), though effective for cancer treatment, can cause immune-related side effects such as inflammatory arthritis (IA) and polymyalgia rheumatica (PMR). This study examined musculoskeletal ultrasound (MSKUS) findings in patients with these ICI-related joint symptoms (MSK-irAEs). Among the 23 patients studied, 83% were diagnosed with MSK-irAEs, and 74% had ultrasound evidence of inflammation. Ultrasound findings led to treatment changes in 70% of cases and helped detect chronic inflammation, which had sometimes been missed for years. Additionally, MSKUS identified other causes of symptoms, supporting ICI continuation in some patients. This study concludes that MSKUS is valuable for both diagnosing and managing ICI-related musculoskeletal issues.

## 1. Introduction

Immune checkpoint inhibitors (ICIs) are immunomodulatory therapies that have transformed cancer treatment, resulting in significant survival benefits across numerous malignancies [1,2]. ICIs were first introduced in 2011 with the development of ipilimumab, an anti-cytotoxic T-lymphocyte-associated antigen-4 (CTLA-4) monoclonal antibody for melanoma [3]. Over the years, the number of available ICIs and their indications has expanded substantially, now including renal, gastric, esophageal, colorectal, lung, and breast cancers, amongst others [4,5,6].

ICIs function by targeting the key regulatory pathways of cytotoxic T-cells, such as CTLA-4, the programmed cell death protein-1 (PD-1)/programmed death ligand-1 (PD-L1) axis, and more recently, lymphocyte activation gene 3 (LAG3), effectively releasing the “brakes” on T-cells and enhancing their ability to eliminate tumor cells [7,8]. However, this non-specific immune activation can trigger a broad range of toxicities known as immune-related adverse events (irAEs) [9,10]. IrAEs can affect nearly any organ system, including multiple at the same time, ranging from the skin to the lungs, and include toxicities resembling rheumatologic diseases [11,12]. Among rheumatologic irAEs, inflammatory arthritis (ICI-IA) and polymyalgia rheumatica-like syndromes (ICI-PMR) are among the most common and persistent manifestations [13,14]. ICI-IA exhibits a wide range of clinical phenotypes, including self-limited oligoarthritis, a spondyloarthritis-like disease, and chronic, erosive arthritis resembling rheumatoid arthritis (RA) [15,16]. While enthesitis and tenosynovitis are distinct from arthritis, most of the literature has grouped the three conditions, when related to ICIs, under the same term of “ICI arthritis”. These manifestations may develop during or after ICI therapy and often persist for months or even years after treatment cessation [15,17].

Despite the increasing recognition of ICI-IA and ICI-PMR, these conditions remain under-characterized, particularly with respect to imaging. Musculoskeletal ultrasound (MSKUS) has become a widely adopted tool for evaluating inflammatory arthritis due to its ability to detect active synovitis, tenosynovitis, enthesitis, and erosions, even in subclinical disease states [18,19,20]. Several small observational studies have begun to explore the role of MSKUS in ICI-IA, providing early insights into disease patterns and burden. For example, Albayda et al. identified MSKUS evidence of synovitis and tenosynovitis in nine patients with new-onset arthritis after ICI therapy, with findings resembling RA in several cases [21]. More recently, Nasrallah et al. examined MSKUS findings in 34 patients with definite ICI-IA, concluding that point-of-care MSKUS is a valuable tool for evaluating potential musculoskeletal irAEs [22]. However, distinct ultrasonographic ICI-IA phenotypes remain under-characterized. Furthermore, the utility of MSKUS in monitoring disease progression or informing treatment decisions, such as corticosteroid tapering, disease-modifying anti-rheumatic drug (DMARD) initiation, or other therapy escalation, has not been fully established.

As more patients live longer with both cancer and chronic ICI-related inflammation, there is an urgent need to better define the imaging features of ICI-IA and ICI-PMR. In this study, we aimed to systematically characterize musculoskeletal US findings in patients with ICI-IA and ICI-PMR and explore their potential role in clinical phenotyping and treatment guidance. For simplicity, we refer to ICI-induced arthritis, enthesitis, tenosynovitis, and PMR collectively as “musculoskeletal irAEs” (MSK-irAEs) throughout this paper.

## 2. Methods

### 2.1. Patient Identification

Eligible patients were identified using Epic’s SlicerDicer tool (https://health.ucdavis.edu/data/epic-slicer-dicer.html). The initial query included all patients with cancer who received ICI therapy between 25 March 2011—the date of ipilimumab’s FDA approval—and 1 April 2025. This cohort was then narrowed to include only those who underwent MSKUS evaluations in the rheumatology clinics at our tertiary care center.

The initial filtering process yielded 187 patients. Study authors conducted comprehensive chart reviews to identify all patients referred specifically for evaluations of inflammatory musculoskeletal diseases potentially related to ICI-based therapy. From this group, a cohort of twenty-two patients was identified as having been referred for MSKUS for suspected MSK-irAEs. An additional patient was captured after the initial query as they underwent MSKUS to evaluate for an MSK-irAE in late April 2025, bringing the final cohort of patients to twenty-three. Demographics, related treatment, and relevant clinical data were extracted from these patients for further descriptive analysis.

This study was approved by the institutional review board at the University of Washington (#00016111).

### 2.2. Patient Classification

Patients were diagnosed with MSK-irAEs based on a combination of MSKUS results, the presence of inflammatory synovial fluid (when available), and a chart review of each patient’s primary rheumatologist’s clinical assessment. The response to therapy was determined by reviewing notes from each patient’s primary rheumatologist while on the therapy. These were categorized into patients with resolved disease, those with improved disease, those in whom it was too early to assess the response to therapy, and patients who were deceased prior to starting therapy following MSKUS. Those deemed “too early to assess response” had either not been followed up with yet after a therapy change or had not had an adequate amount of time with which to assess the response.

### 2.3. Musculoskeletal Ultrasound Evaluation

All patients were referred for an MSKUS examination by their consulting rheumatologist at the University of Washington Medical Center. The MSKUS investigations were requested to further clarify the etiology of the patient’s musculoskeletal pains. All patients were clinically suspected of or previously diagnosed as having an MSK-irAE. Depending on the clinical presentation, one of the following US exams was performed: the standard European League Against Rheumatism/American College of Rheumatology (EULAR/ACR) protocol on symptomatic and contralateral joints for comparison, the ARCTIC (modified) synovitis examination of involved joints, or enthesitis examinations (Madrid Sonographic Enthesitis Index (MASEI) protocol) [23,24,25]. When clinically appropriate, joint aspirations or steroid injections were performed.

MSKUS studies were performed on a GE LOGIQ P9 US machine with the grayscale setting optimized for each joint, using the highest frequency to optimize the joints being evaluated. The L 4–12 MHz linear phased array transducer (General Electric, Boston, MA, USA) was used for deeper joints, and the L8-18i MHz hockey stick transducer was used for more superficial joints [26]. Power Doppler settings were adjusted to accurately evaluate low flow vascularity, with a pulse repletion frequency (PRF) of 0.6mHz and gain adjusted per ACR/EULAR recommendations [27].

All MSKUS examinations were performed by one of two rheumatologists certified in MSKUS (Examiner 1—American Registry for Diagnostic Medical Sonography (ARDMS)-certified with 20 years of experience or Examiner 2—Musculoskeletal Ultrasound Certification in Rheumatology (RhMSUS)-certified with 12 years of experience). Individual joints were scanned in 2 orthogonal planes (long and short axis) following the standard scanning protocols recommended by ACR/EULAR [26]. The findings were defined according to published Outcome Measures in Rheumatoid Arthritis Clinical Trials (OMERACT) definitions, while a semiquantitative scoring system was used for synovial hypertrophy and power Doppler according to EULAR/OMERACT systems [28]. The joints were assessed for the presence of synovial hypertrophy, effusion, and power Doppler, the tendons were assessed for edema, calcifications, enthesopathy, and power Doppler, and the bone cortical surfaces were assessed for erosions and osteophytes.

The patients’ MSKUS findings were categorized with the following novel scoring system: 0—no signs of inflammatory arthropathy or tendinopathy, 1—potential signs of inflammation (grayscale ≥ 2, effusion without power Doppler, synovial hypertrophy in the joint), and 2—active inflammation in joints and/or tendons (characterized by power Doppler) and signs of inflammation. MSKUS images were reviewed independently by both certified examiners with 100% inter-examiner reliability.

### 2.4. Statistical Analysis

Descriptive statistics were performed and calculated in Microsoft Excel for the entire patient cohort to summarize demographic, clinical, and treatment-related variables. Measures of central tendency and dispersion (e.g., means, medians, and standard deviations) were used for continuous variables, while categorical variables were summarized using frequencies and percentages.

## 3. Results

### 3.1. Demographic and Clinical Characteristics

Demographic and clinical data of the 23 included patients are presented in Table 1. The mean age of patients was 61 years while the median was 62 years, with most patients being male (*n* = 12, 52%). Most patients (*n* = 20) were White, while the remainder were Asian (*n* = 3). Melanoma was the most common malignancy, accounting for 48% (*n* = 11) of cases, with three cases each of renal-cell carcinoma and breast cancer, two cases each of lung cancer and urothelial carcinoma, and a single case each of endometrial carcinoma and neuroendocrine carcinoma. PD-1 inhibitors were the most common ICI used, with fifteen patients (65%) receiving them. Other patients received either combination therapy with CTLA-4 and PD-1 or PD-1 and LAG3, or monotherapy with PDL-1 ICIs. Additional immune-related adverse events (irAEs) were identified in 48% of patients (*n* = 11).

Most patients (*n* = 15) developed symptoms concerning MSK-irAEs while actively receiving an ICI, and the remainder (*n* = 8) developed them following therapy cessation or completion. The mean (SD) number of ICI cycles prior to symptom onset was 7.7 months (6.92), with a wide range observed. The mean (SD) time from symptom onset to MSKUS was 12.74 (17.9) months, while the median (IQR) time was 5 (14.5) months. Median follow-up from MSKUS in those diagnosed with an MSK-irAE was 7 months, with a range of 1–20 months.

Nine patients underwent MSKUS while still actively receiving ICI therapy. Seventeen patients received treatment for MSK-irAE prior to their US, though only eleven remained on MSK-irAE-directed therapy at the time of imaging. Oral steroids were the most common treatment prior to US (*n* = 17). Three patients received intra-articular steroid injections, seven received csDMARDs, and two received biologics. At the time of US, eight patients were on steroid monotherapy, two were on a combination of csDMARDs and steroids, and one was receiving a biologic. Twelve patients were on no therapy at the time of their US.

### 3.2. Diagnosis and Ultrasound Findings

**Figure 1:**
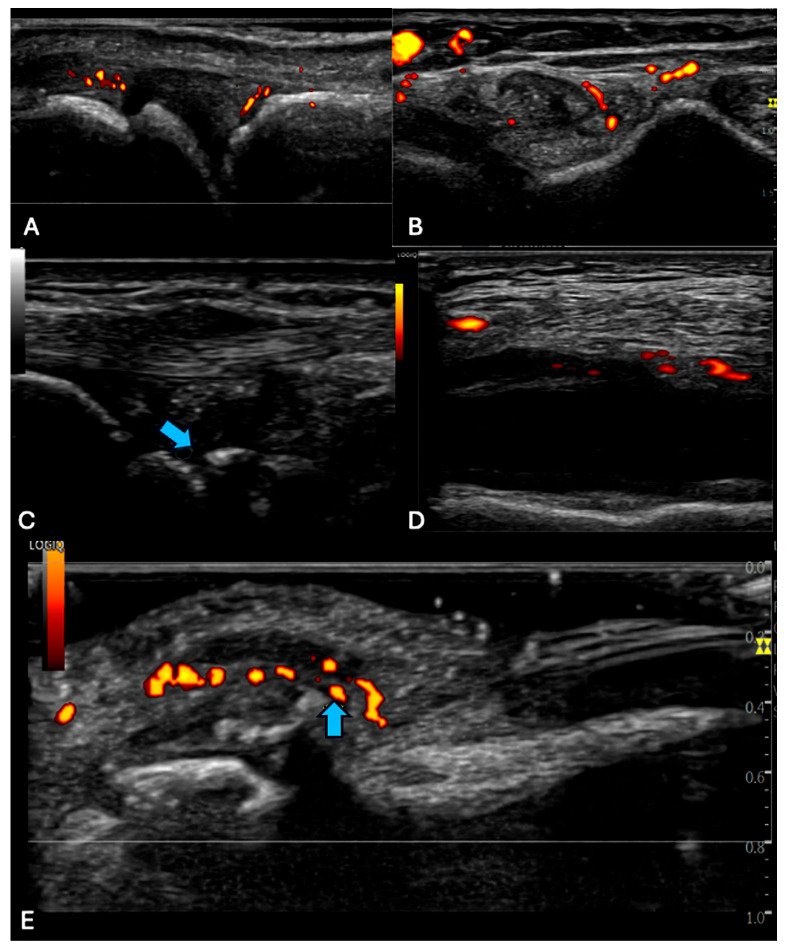
Representative ultrasound images showing (**A**) MCP synovitis, long axis, (**B**) tenosynovitis of the wrist, short axis, (**C**) erosion of the lunate, wrist, long axis (arrow), (**D**) transverse suprapatellar recess with effusion, synovial hypertrophy, and power Doppler, short axis, and (**E**) enthesitis DIP extensor tendon, long axis (arrow).

Representative US findings are depicted in Figure 1, while the results of patients’ US are expressed in Table 2. In total, 83% of patients (*n* = 19) were diagnosed with an MSK-irAE. Seventeen patients had signs of inflammation on their ultrasound, with scores of either 1 or 2, while two patients scored a 0. Fourteen patients exhibited active joint inflammation, most frequently affecting the knees (*n* = 9), followed by the hands, including metacarpophalangeal joints (MCP), proximal interphalangeal joints (PIP), and distal interphalangeal joints (DIP), as well as ankles, wrists, and elbows.

US findings are categorized with the following scoring system: 0—no signs of inflammatory arthropathy or tendinopathy, 1—potential signs of inflammation (grayscale ≥ 2, effusion without power Doppler, synovial hypertrophy in the joint), and 2—active inflammation in joints and/or tendons (characterized by power Doppler) and signs of inflammation.

Tendon involvement (tenosynovitis or enthesitis) secondary to an MSK-irAE was present in eight patients. An additional patient had DeQuervain’s tenosynovitis that was deemed to be unrelated to their MSK-irAE. This patient was diagnosed with an MSK-irAE primarily based on their clinical course. Inflammatory tendon findings occurred at the patellar tendon, distal quadriceps tendon, posterior tibial tendon, wrist extensors, biceps tendon, distal interphalangeal (DIP) extensor, metacarpophalangeal (MCP) extensor, Achilles tendon, plantar aponeurosis, extensor pollicis brevis, abductor pollicis longus, and triceps tendon.

Five patients had evidence of inflammation in both joints and tendon structures. Effusions were identified most commonly in knees and were also seen in ankles, wrists, and hands. Synovial fluid analysis was available for six patients with MSK-irAEs, all of which were inflammatory and from the knee joints. MSKUS-detected erosions were present in four patients with MSK-irAEs and one patient with erosive osteoarthritis (OA). Enthesophytes were detected in a single patient with an MSK-irAE, as well as in one patient with non-inflammatory pain.

### 3.3. Therapeutic Changes Following Ultrasound

Information on therapeutic changes is shown in Table 1. Following MSKUS, sixteen patients had therapy escalations, including oral steroids (*n* = 5), intra-articular steroid injections (*n* = 7), csDMARDs (*n* = 7), and biologic therapy (*n* = 1), while one patient remained undecided between csDMARDs or biologic therapy at the time of data collection (Table 3). A patient who was found to have diffuse enthesitis was switched from rituximab to adalimumab, with no response to the former; an assessment of response to the latter is ongoing. Another patient had inflammation upon MSKUS; however, she elected to enter hospice care and passed away prior to initiating any new therapy for her MSK-irAE. Four patients required additional therapeutic alterations beyond their initial post-US treatment.

Regarding outcomes following therapy changes, five patients achieved the complete resolution of MSK-irAE symptoms, and nine patients demonstrated clinical improvements at follow-up. Three patients had only recently initiated new therapies and had not yet been assessed for their response, while one patient passed away prior to evaluation. One patient was still deciding on the therapy with which to address their MSK-irAE. Three patients had their ICI halted due to concerns of an MSK-irAE prior to US. One of these patients resumed their ICI after their MSKUS findings revealed carpal tunnel syndrome rather than an MSK-irAE, while two patients remained off therapy.

## 4. Discussion

In our small cohort of 23 patients who underwent MSKUS for evaluation of MSK complaints following ICI treatment initiation, MSKUS confirmed MSK-irAEs in 83% of patients (*n* = 17), most commonly showing joint and tendon inflammation, effusions, and erosions. Following imaging, sixteen (74%) patients received therapy escalation (steroids, csDMARDs, or biologics), with most achieving symptom improvement. Immune checkpoint inhibitor (ICI) therapy had been discontinued in three cases prior to MSKUS, although one patient later resumed treatment after re-evaluation.

With the expanding use of ICIs in cancer treatment, rheumatic irAEs are being reported with greater frequency. Among these, ICI-IA and ICI-PMR are among the most prevalent. ICI-IA presents with significant heterogeneity in terms of anatomical involvement, disease burden, and chronicity. A critical clinical task is to distinguish true MSK-irAEs from non-inflammatory causes of musculoskeletal pain. This differentiation is essential for several reasons: it enables the timely initiation of appropriate treatments to minimize patient pain and disability, prevents the unnecessary use of immunosuppressive agents, and helps avoid the premature discontinuation of ICIs, which may otherwise compromise oncologic outcomes.

Importantly, our study highlights the clinical utility of MSKUS not only as a diagnostic tool but also to help guide therapeutic decision-making. Nearly all patients with active inflammation on their MSKUS underwent changes in management, except for one individual who transitioned to hospice care. One notable case involved a patient with diffuse enthesitis on imaging and refractory symptoms despite the prior use of steroids, methotrexate, and rituximab. The MSKUS findings suggested a peripheral spondyloarthritis-like phenotype, prompting the initiation of adalimumab. Overall, most patients who received MSK-irAE treatment modifications based on MSKUS findings experienced symptomatic improvement or resolution, suggesting a role for MSKUS in facilitating targeted, individualized care. MSKUS also helped unearth other pathologies of pain and/or rule out MSK-irAEs in several of our patients, preventing the initiation of unneeded immunosuppressive medications, as well as allowing for ICI resumption in one patient. These findings suggest the integration of MSKUS into the diagnostic and therapeutic algorithm for patients with suspected MSK-irAEs, with the potential to improve outcomes through earlier interventions and more precise treatment selection.

Our findings are consistent with prior studies reporting the broad spectrum of MSKUS findings in these pathologies, which can include synovitis, tenosynovitis, enthesitis, and erosive disease [21,22]. Within our cohort, the knee was the most frequently affected joint and the most aspirated site for inflammatory synovial fluid. Tendinous involvement, including tenosynovitis and enthesitis, was also commonly observed. Structural changes, such as erosions and enthesophytes, were present in several patients. Notably, individuals with erosive changes often experienced prolonged intervals between symptom onset and MSKUS evaluation—ranging from 17 to 82 months—suggesting that such findings may reflect chronic, under-recognized inflammation. In RA, for comparison, erosive damage starts within and is the most rapid during the first five years of diagnosis, with undertreated or untreated disease leading to often permanent harm [29,30]. These observations underscore the need for early recognition and monitoring of MSK-irAEs to prevent irreversible joint damage.

Despite the valuable insights provided by our study, several limitations should be acknowledged. First, the retrospective design and reliance on chart review inherently introduce several potential biases, including variability in documentation and missing clinical details. Our sample may not be fully representative of the MSK-irAE population as a whole, as rheumatology providers may also differ in how likely they are to refer a patient for MSKUS. MSKUS also follows specific protocols (i.e., ARCTIC or MASEI) depending on the presumed affected joints and may miss inflamed but clinically silent joints or entheses. Additionally, numerous patients had already been initiated on therapy prior to undergoing MSKUS, which may have influenced the imaging findings, clinical outcomes, and subsequent clinical decisions. Finally, the relatively small sample size in a single tertiary center significantly limits the generalizability of our conclusions and reduces the statistical power to pursue further analyses. Therefore, our findings are hypothesis-generating and require further external validation in larger studies.

Looking into the future, additional research is needed to clarify the role of MSKUS in the clinical phenotyping of MSK-irAEs, such as rheumatoid arthritis-like versus spondyloarthritis-like disease, and determine how specific imaging patterns, such as tendon vs. joint involvement, may inform personalized treatment strategies. Such work could support more nuanced therapeutic algorithms based on imaging-guided classification. In parallel, broader access to MSKUS remains an important goal, as its availability is currently constrained by the small supply of providers with specialized training in this modality, their lack of administrative support, and their limited time to perform such tests [31]. Expanding training opportunities and incorporating MSKUS into routine rheumatologic evaluation may enhance diagnostic precision and optimize patient care.

## 5. Conclusions

Our study highlights the clinical utility of MSKUS not only as a diagnostic tool but also to guide therapeutic decision-making.

## Figures and Tables

**Table 1:** Demographic and clinical features of the cohort undergoing MSKUS at our center.

Category	Variable	Value (Percentage)
Patients	Number of Patients	*n* = 23
Age	Median	62
Sex	Male	12 (52.2%)
	Female	11 (47.8%)
Race	White	20 (87%)
	Asian	3 (13%)
Malignancy type	Melanoma	11 (47.8%)
	RCC	3 (13%)
	Lung Cancers	2 (8.7%)
	Breast Cancers	3 (13%)
	Urothelial Carcinoma	2 (8.7%)
	Endometrial Carcinoma	1 (4.3%)
	Neuroendocrine carcinoma	1 (4.3%)
ICI Type	PD-1	15 (65.2%)
	PDL-1	1 (4.3%)
	PD-1 + CTLA-4	4 (17.4%)
	PD-1 + LAG3	3 (13%)
Additional irAEs	Mean	1.1
	Median	1
	Number of Patients with other irAEs	11 (47.8%)
	Number of Patients without other irAEs	12 (52.2%)
Symptom Timing	On ICI	15 (65.2%)
	Post-ICI	8 (34.8%)
ICI Cycles prior to Symptoms	Mean	7.7
	Median	7
Duration of Time between Symptoms and MSKUS	Mean Months	12.74
	Median Months	5
MSKUS Timing in Relation to ICI	On ICI	9 (39.1%)
	Post-ICI	14 (60.9%)
MSK-irAE Therapy Initiation	On Therapy Prior to MSKUS	17 (73.9%)
	No Therapy Prior to MSKUS	6 (26.1%)
MSK-irAE Therapy at Time of MSKUS	On Therapy	11 (47.8%)
	Not on Therapy	12 (52.2%)
MSK-irAE Therapies Prior to MSKUS	Steroid (PO)	17 (73.9%)
	CSI	3 (13%)
	csDMARD	7 (30.4%)
	Biologic	2 (8.7%)
	None	6 (26.1%)
MSK-irAE Therapy Type at MSKUS	Steroid (PO) Monotherapy	8 (34.8%)
	csDMARD + Steroid (PO)	2 (8.7%)
	Biologic	1 (4.3%)
	None	12 (52.2%)
Final Diagnosis	MSK-irAE	19 (82.6%)
	No MSK-irAE	4 (17.4%)
Escalation in MSK-irAE Therapy Post-MSKUS	Therapy Escalation	16 (69.6%)
	No Therapy Change	7 (30.4%)
Additional Therapy Type (n = 16)	Steroid (PO)	5 (31.3%)
	CSI	7 (43.8%)
	csDMARD	7 (43.8%)
	Biologic	1 (6.3%%)
	Patient Deciding	1 (6.3%%)
Future MSK-irAE therapy Changes (in those with MSK-irAEs, n = 19)	Further Therapies	4 (21.1%)
	No Further Therapies	15 (78.9%)
Response to Therapy in those with MSK-irAEs (n = 19)	Resolved MSK-irAE	5 (26.3%)
	Improved MSK-irAE	9 (47.4%)
	Too early to evaluate	4 (21.1%)
	Patient deceased prior to Evaluation	1 (5.3%)
Follow-up from MSKUS in those with MSK-irAEs (months)	Median	7
ICI Halted due to concern for MSK-irAE	Yes	3 (13%)
	No	20 (87%)
Changes in ICI Following MSKUS (for those on ICI at time of MSK-irAE, occurrence n = 15)	Halted	2 (13.3%)
	Resumed	1 (6.7%)
	No Change	12 (80%)

Abbreviations: CSI: corticosteroid injection; PO: per os; csDMARD: conventional synthetic disease-modifying anti-rheumatic drug; RCC: renal-cell carcinoma; ICI: immune checkpoint inhibitor; CTLA-4: cytotoxic T-lymphocyte-associated protein 4; PD-1: programmed cell death protein 1; PDL-1: programmed death ligand 1; MSKUS: musculoskeletal ultrasound; irAE: immune-related adverse event; MSK-irAE: musculoskeletal immune-related adverse event.

**Table 2:** Ultrasound features.

Pt	Scoring	Location	Joint(s)			Bone	Tendon Involvement			Inflammatory Synovial Fluid (1: Yes; 2: No; -: No Fluid)	Final Diagnosis
			GS	PD	Effusion	Erosions	TS	En	Enp		
UW1	1	Knee	2	-	+	-	-	-	-	1	MSK-irAE
		Ankle	1	-	+	-	-	-	-	-	
UW2	0	Hand	2	1	-	+	-	-	-	-	Severe OA
UW3	1	Knee	2	-	+	-	-	-	-	1	MSK-irAE
UW4	2	Knee	2	1	+	-	-	+ (patellar tendon)	-	-	MSK-irAE
		Ankle	2	1	+	-	+ (posterior tibial tendon)	-	-	-	
UW5	2	Knee	2	2	+	-	+ (quadriceps tendon)	-	-	1	MSK-irAE
UW6	0	Knee	-	-	+	-	-	-	-	-	Non-inflammatory pain
UW7	0	Hand	1	-	-	-	-	-	-	-	MSK-irAE
UW8	2	Knee	2	-	+	-	-	-	-	-	MSK-irAE
		R Wrist	1	1	-	-	+ (extensor tendons)	-	-	-	
		L Wrist	-	1	-	-	-	-	-	-	
		L 3rd MCP	-	1	-	-	-	-	-	-	
		Ankle	1	-	+	-	-	-	-	-	
UW9	1	Knee	2	-	+	-	-	-	-	1	MSK-irAE
UW10	2	Knee	2	1	+	-	-	-	-	1	MSK-irAE
UW11	2	R Wrist	2	1	-	+	-	+ (extensor tendons)	-	-	MSK-irAE
		L Wrist	2	2	-		-	+ (extensor tendons)	-	-	
		R 2nd MCP	1	-	-		-	-	-	-	
		L 2nd MCP	1	-	-		-	-	-	-	
UW12	2	Hand	-	1	-	-	-	+ (DIP extensor tendon enthesitis)	-	-	MSK-irAE
UW13	0	Ankle	-	-	-	-	-	-	+	-	Non-inflammatory pain
		Foot	-	-	+	-	-	-	+	-	
UW14	1	Shoulder	-	-	-	-	+ (L biceps tendon)	-	-	-	MSK-irAE
UW15	2	Knee	2	1	+	-	-	-	-	-	MSK-irAE
UW16	2	Knee	-	-	-	+	-	+ (quadriceps tendon enthesitis, patellar tendon enthesitis)	+	-	MSK-irAE
		Ankle	-	-	-		-	+ (Achilles tendon enthesitis)	+	-	
		Foot	-	-	-		-	+ (plantar aponeurosis enthesitis)	-	-	
		Elbow	-	-	-		-	+ (triceps enthesitis)	+	-	
UW17	2	R Hand	1	1	+	+	+ (2nd and 3rd MCP extensor tendons)	-	-	-	MSK-irAE
		L Hand	1	-	+	-	-	-	-	-	
UW18	2	R Hand	1	1	-	-	-	-	-	-	MSK-irAE
		L Hand	1	1	-	-	-	-	-	-	
UW19	0	Wrist	-	-	-	-	+ (R extensor pollicis brevis and abductor pollicis longus)	-	-	-	MSK-irAE
UW20	0	Hand	1	-	-	-	-	-	-	-	Carpal Tunnel Syndrome
UW21	2	R Wrist	2	1	+	+	+ (extensor tendons)	-	-	-	MSK-irAE
		L Wrist	2	1	+		+ (extensor tendons)	-	-	-	
UW22	2	R Knee	2	1	+	-	-	-	-	1	MSK-irAE
UW23	2	Hand	2	2	+	-	-	-	-	-	MSK-irAE

Abbreviations: GS: grayscale; PD: power Doppler; TS: tenosynovitis; En: enthesopathy; Enp: enthesophyte; R: right; L: left; MCP: metacarpal; DIP: distal interphalangeal joint; Pt: patient.

**Table 3:** irAE-targeted therapies in our patient cohort.

Pt	Anti-Inflammatory Therapy at Time of MSKUS	Past Treatments for MSK-irAEs	Change in MSK-irAE Therapy Following US?	Change	Additional MSK-irAE Treatments (Outside of Immediate Changes)
UW1	None	None	Yes	CSI	Prednisone, Tocilizumab
UW2	None	Prednisone, MTX, HCQ	No	NA	NA
UW3	Prednisone, MTX	Prednisone	Yes	CSI, MTX increase	NA
UW4	Prednisone	Prednisone	Yes	Prednisone increase	NA
UW5	Prednisone	Prednisone	Yes	CSI, Prednisone increase	MTX
UW6	None	None	No	NA	Prednisone, HCQ, SSZ
UW7	None	Prednisone, SSZ	Yes	Remained off treatment	Prednisone, HCQ
UW8	Prednisone	Prednisone	Yes	MTX	NA
UW9	None	None	Yes	CSI	None
UW10	Prednisone	Prednisone, HCQ, CSIs, MTX, Tocilizumab	Yes	CSI	Tocilizumab, CSIs, Prednisone
UW11	None	None	Yes	Prednisone, MTX	None
UW12	None	None	Yes	Prednisone	None
UW13	None	Prednisone	No	NA	None
UW14	Prednisone	Prednisone	No	NA	None
UW15	Prednisone	Prednisone	Yes	MTX	None
UW16	Rituximab	Prednisone, MTX, Rituximab	Yes	Adalimumab	None
UW17	None	Prednisone	Yes	HCQ	None
UW18	Prednisone	Prednisone, MTX	No	NA	None
UW19	Prednisone, MTX	Prednisone, MTX	No	NA	None
UW20	None	None	No	NA	None
UW21	None	Prednisone, CSIs	Yes	CSI, prednisone, MTX	None
UW22	Prednisone	Prednisone, CSI	Yes	CSI, MTX	None
UW23	None	Prednisone	Yes	Deciding	None

Abbreviations: CSI: corticosteroid injection; MSKUS: musculoskeletal ultrasound; irAE: immune-related adverse event; MSK-irAE: musculoskeletal immune-related adverse event; MTX: methotrexate; HCQ: hydroxychloroquine; SSZ: sulfasalazine; NA: not applicable.

## Data Availability

The original contributions presented in this study are included in the article. Further inquiries can be directed to the corresponding author.
